# Mapping DNA conformations and interactions within the binding cleft of bacteriophage T4 single-stranded DNA binding protein (gp32) at single nucleotide resolution

**DOI:** 10.1093/nar/gkaa1230

**Published:** 2020-12-24

**Authors:** Benjamin R Camel, Davis Jose, Katarina Meze, Anson Dang, Peter H von Hippel

**Affiliations:** Institute of Molecular Biology and Department of Chemistry and Biochemistry, University of Oregon, Eugene, OR 97403, USA; Institute of Molecular Biology and Department of Chemistry and Biochemistry, University of Oregon, Eugene, OR 97403, USA; Department of Chemistry and Physics, Monmouth University, West Long Branch, NJ 07764, USA; Institute of Molecular Biology and Department of Chemistry and Biochemistry, University of Oregon, Eugene, OR 97403, USA; Institute of Molecular Biology and Department of Chemistry and Biochemistry, University of Oregon, Eugene, OR 97403, USA; Institute of Molecular Biology and Department of Chemistry and Biochemistry, University of Oregon, Eugene, OR 97403, USA

## Abstract

In this study, we use single-stranded DNA (oligo-dT) lattices that have been position-specifically labeled with monomer or dimer 2-aminopurine (2-AP) probes to map the local interactions of the DNA bases with the nucleic acid binding cleft of gp32, the single-stranded binding (ssb) protein of bacteriophage T4. Three complementary spectroscopic approaches are used to characterize these local interactions of the probes with nearby nucleotide bases and amino acid residues at varying levels of effective protein binding cooperativity, as manipulated by changing lattice length. These include: (i) examining local quenching and enhancing effects on the fluorescence spectra of monomer 2-AP probes at each position within the cleft; (ii) using acrylamide as a dynamic-quenching additive to measure solvent access to monomer 2-AP probes at each ssDNA position; and (iii) employing circular dichroism spectra to characterize changes in exciton coupling within 2-AP dimer probes at specific ssDNA positions within the protein cleft. The results are interpreted in part by what we know about the topology of the binding cleft from crystallographic studies of the DNA binding domain of gp32 and provide additional insights into how gp32 can manipulate the ssDNA chain at various steps of DNA replication and other processes of genome expression.

## INTRODUCTION

The single-stranded DNA binding protein of bacteriophage T4 (gp32) plays a central role in the function of the T4 DNA replisome. It binds cooperatively in linear clusters or filaments to protect the single-stranded DNA (ssDNA) templates exposed by the helicase-primosome from nuclease attack, to prevent the formation of local ssDNA secondary structure in these templates and to ‘lay-out’ these template strands into an optimal (extended) conformation for copying by the T4 DNA polymerase. Gp32 also plays a coordinating role in integrating the activities of the three ‘functional sub-assemblies’ of the replication complex: (i) the helicase-primase complex (the primosome); (ii) the leading- and lagging-strand DNA polymerases; and (iii) the clamp-clamp loader complex that controls the processivity of the DNA synthesis catalyzed by the polymerases. In addition, gp32 is involved in controlling the interactions and activities of other regulatory proteins during the various steps of the DNA replication cycle (1–3).

The gp32 monomer is a 33.5 kDa protein with three distinct domains: (i) a central single-stranded DNA (ssDNA) binding core that spans seven ssDNA nucleotide residues (nts); (ii) a short (and at least partially unstructured) N-terminal ‘tail’ sequence that controls binding cooperativity; and (iii) a longer (and likely initially largely unstructured) C-terminal sequence that modulates interactions with other T4-coded proteins involved in regulation of replication and related processes of genome expression (1,4). The DNA binding core binds the negatively charged sugar-phosphate backbone of the template ssDNA into an extended electropositive binding cleft (5). This cleft is lined with positively charged amino acid residues that facilitate largely sequence-independent ssDNA binding via favorable electrostatic interactions with the backbone phosphates of the ssDNA templates exposed by the T4 helicase-primosome, and also with aromatic amino acid residues that may partially stack between (or otherwise interact with) the ssDNA bases (5).

In its initial binding to ssDNA as monomer subunits, gp32 binds most tightly to the DNA backbone at 2–3 nt positions located near the 3′-end of the binding footprint (6). At higher gp32 concentrations the protein shifts into a cooperative binding mode, in which the N-terminal domain promotes the cooperative binding of gp32 monomers to the ssDNA (7,8). The C-terminal domain, which is also known as the ‘acidic’ domain because of its high content of negatively charged amino acid residues, plays the major role in the interactions of ssDNA-bound gp32 molecules with the other regulatory proteins of replication (and recombination and repair) (9–11).

Previous studies from our laboratory, using 2-AP monomer and dimer base-analogue substituents as spectroscopic probes, have examined the binding, as isolated monomers, of gp32 molecules to short ssDNA lattices (8 nts in length) (6), and also as contiguously and cooperatively bound gp32 clusters to longer lattices (25 or more nts in length) (12). The binding affinities of gp32 in both binding modes are largely independent of the base composition and sequence of the lattice (13), supporting the notion that gp32 binding in both modes primarily involves direct interactions of the residues of the binding cleft with the sugar-phosphate backbone of ssDNA. Gp32 binds to oligo(dT)_n_ sequences with a slightly higher affinity than it does to other homo-oligomer ssDNA sequences (14,15). This is consistent with the observation that cooperative gp32 binding extends the ssDNA lattice (16), resulting in unstacking of the ssDNA bases. And because ssDNA dT sequences are somewhat less stacked than others, less binding free energy needs to be dissipated in the unstacking component of the binding process than for other base sequences. This makes oligo(dT)-containing lattices particularly useful in ‘foot-printing’ analyses of gp32–ssDNA interactions.

Here, we focus on the interactions of the gp32 binding cleft with ssDNA lattices by studying – at single nucleotide resolution – the binding ‘footprint’ of cooperatively bound gp32 monomers to oligo(dT)_*n*_ ssDNA lattices containing site-specifically positioned 2-aminopurine (2-AP) probes. 2-AP is an analogue of adenine, differing only from the canonical base in the translocation of the 6-amino Watson-Crick H-bonding donor of the adenine ring to the 2-amino position. This shifts the ‘outer’ H-bond of the Watson–Crick base pair from the major groove to the minor groove, with no significant change in the stability of the base-paired duplex. The 2-AP analogue is fluorescent, and has been extensively used in DNA–protein interaction studies because it generally produces only minimal functional perturbation of protein interactions that occur with the same DNA sequences containing adenine bases (17–19). This probe is an especially useful spectroscopic tool because the fluorescence and circular dichroism (and absorbance) spectra of these base analogues can be excited and detected at wavelengths greater than 300 nm, where the canonical nucleotide bases of DNA (and the amino acid residues of proteins) are optically transparent (20,21).

We use the 2-AP spectroscopic probe in three complementary ways to map the environments and interactions of nucleotide bases at each position in the gp32 binding cleft under saturating (cooperative) binding conditions. (i) The fluorescence of 2-AP is significantly quenched by neighboring bases through nearest neighbor base-base stacking interactions, as well as by changes in the polarity of the local environment and by changes in the dielectric constant of the solvent environment (12). These properties permit us to monitor changes in stacking interactions (relative to the fluorescence of the probes in the unbound ssDNA construct) at each position in the cleft as a consequence of gp32 binding, as well as local effects induced by nearby amino acid residue polarity differences within the cleft. (ii) The fluorescence of 2-AP probes can also be perturbed by interactions with collisional (Stern-Volmer) quenching agents added to the solvent. Here we add increasing concentrations of acrylamide monomers to the solution to act as uncharged quenchers of the fluorescence of 2-AP probes located in each position of the bound ssDNA construct, which permits us to discriminate these specifically-positioned 2-AP probes in terms of the changes in their access to the solvent environment as a consequence of gp32 binding. (iii) Finally, circular dichroism measurements can be used to study changes in exciton coupling within pairs of adjacent 2-AP probes and therefore also reflect—in a still different way—local conformational changes in the ssDNA with gp32 addition at each binding position within the gp32 cleft. We use these approaches to define local changes in the conformations of the ssDNA lattice as a function of probe position within the ssDNA binding cleft that occur as a consequence of gp32 binding, as well as to monitor the assembly of the cooperatively bound gp32 clusters on ssDNA lattices as these constructs are titrated with increasing concentrations of gp32. These approaches have permitted us to begin to map, at single nt resolution, the interactions of ssDNA within the gp32 binding cleft under various environmental conditions.

## MATERIALS AND METHODS

### DNA constructs and nomenclature

Unlabeled and 2-aminopurine (2-AP) labeled DNA oligonucleotides were purchased from (and manufactured by) Integrated DNA Technologies (IDT) (Coralville, IA, USA). All oligos had -OH groups at both the 3′ and the 5′ ends of the ssDNA chains. Lyophilized DNA was re-suspended in experimental buffer (described below) and concentrations were determined by UV absorbance at 260 nm, using extinction coefficients furnished by the manufacturer. The sequences and nomenclature of the DNA constructs used in this study are listed in Table 1.

**Table 1.** Nomenclature and structures of 2-AP-containing ssDNA constructs. Oligo-(dT) single-stranded DNA constructs site-specifically labeled with 2-AP monomer or dimer-pair probes (shown as red X’s). The sequence notations that define these constructs use a preceding subscript to denote the length of the ssDNA lattice, followed by T and then by a following subscript to indicate the position(s) of the 2-AP probe(s) relative to the 5′-end of the ssDNA lattice

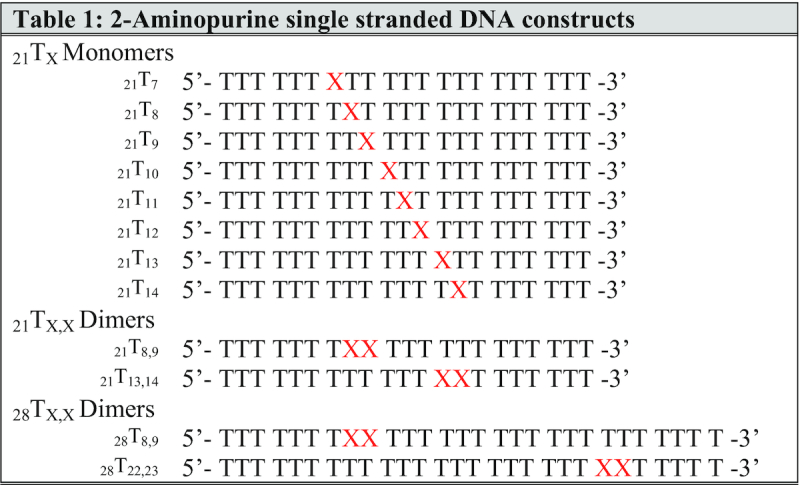

### Cell growth, protein purification and buffers used

pYS6/AR120 cells were grown to an optical density (OD_600_) of 0.9–1.0 at 37°C in Luria-Bertani liquid medium (LB broth) containing 50μg/ml ampicillin. The cells were then induced by adding nalidixic acid to a final concentration of 40 μg/ml, grown for an additional 8–10 h at 37°C and harvested. The gp32 protein was purified according to the procedure of Bittner *et al.* (22) and stored at −80°C in storage buffer containing 20 mM Tris–OAc (pH 8.1), 0.5 mM DTT, 1 mM EDTA, 50 mM KOAc and 10% glycerol. All experiments were performed at 20°C in ‘experimental buffer’ containing 10 mM 4-(2-hydroxyethyl)-1-piperazine-ethanesulfonic acid (HEPES) and 30 mM potassium acetate (KOAc) at pH 7.5.

### Spectroscopic measurements

The total concentrations of ssDNA oligomer and protein were calculated using UV–visible absorbance at 260 and 280 nm, respectively. Spectra were obtained with a Varian Cary 3E UV–Visible spectrophotometer using samples in 1 cm path-length quartz cuvettes. CD spectra were measured in 1 cm path-length optical grade quartz cells over the wavelength range 300–400 nm, using JASCO model J-720 and J-810 CD spectrometers. For each spectrum shown in our figures, 20 individual data sets were collected at a bandwidth of 0.5 nm and a scanning rate of 50 nm/min. The experiments were performed at a DNA lattice concentration of either 6 or 3 μM and the total concentrations of ssDNA oligomer and gp32 at each point in the titrations were calculated from the added volumes and input concentrations of gp32 monomers per binding site. Fluorescence measurements were performed in 4 × 4 mm optical quartz cells in a Horiba FluoroMax-4 spectrophotometer. Samples were excited at 315 nm, and emission spectra were collected from 330 to 450 nm. Samples containing DNA constructs were titrated with increasing amounts of concentrated gp32 (40 μM) or acrylamide (1 M) stock solutions. In all experiments the samples were gently mixed, equilibrated for two minutes and scanned at a constant temperature of 20°C. Corrections for protein based or background fluorescence were made by subtracting the fluorescence intensities obtained with an unlabeled ssDNA construct of the same length and titrating with the same amounts of gp32 as used in the experiments with probe-containing constructs.

### Data analysis

The CD spectra replicates were averaged and plotted as graphs of Δϵ/AP (M^−1^ cm^−1^) as a function of wavelength. Raw fluorescence data were corrected for background counts and dilution, and for spectral contributions from ssDNA and gp32. Gp32 titration data monitored at the fluorescence maxima of 370 nm were normalized to a fluorescence ratio (*F/F*_max_) of 1.0, where *F* is the fluorescence at each point in the titration and *F*_max_ is the maximum fluorescence value attained in the titration. To obtain binding stoichiometry, individual linear extrapolations of the first six (slope) and last six (plateau) data points were calculated for each titration. The intercepts of these two linear extrapolations were used to establish the concentration at which the construct is fully saturated with gp32 in each titration. The uncertainty of the measurements was estimated by the standard deviation of the calculated equivalence points from individual replicated titrations. The maximal fluorescence increase upon binding, for each replicate set of titrations, was calculated as the ratio of the fluorescence intensity for the ssDNA construct fully saturated with protein $({F_P})$ and the fluorescence intensity of the construct in the absence of protein $({F_0})$.

Acrylamide fluorescence quenching data were plotted as ratios of the fluorescence intensity in the absence of quencher $({F_0})$over the intensity (*F*) after subsequent additions of quencher (*Q*) (23). The data points showed a clear linear trend and were fit by linear regression to obtain ${K_{SV}}$, the Stern-Volmer quenching constant, using the Stern-Volmer equation written as follows:

(1) $$\begin{equation*}{K_{SV}} = \frac{{{F_0}}}{F} = 1 + {K_D}\left[ Q \right]\end{equation*}$$

Here, *K*_D_ is the Stern–Volmer quenching constant (for collisional quenching) and [Q] is the quencher concentration (M) (here monomeric acrylamide). We note that *K*_D_^−^^1^ is the quencher concentration at which $\frac{{{F_0}}}{F} = 2$ (i.e. the concentration at which 50% of the initial fluorescence has been quenched). In general, especially if the quenching mechanism(s) is/are not fully understood, the ‘apparent’ quenching constant is written as *K*_SV_, and corresponds to the slope of the Stern-Volmer plot obtained by linear regression. All linear regressions and the accompanying statistics were generated using GraphPad Prism 8 plotting and fitting software. Error bars for each data point represent the standard deviation of three to six repeats of the relevant measurement.

## RESULTS

### Gp32 binds cooperatively and stoichiometrically to both 21-mer and 28-mer 2-AP probe-labeled oligo(dT) lattices

Previous studies from our lab have utilized the increase in the fluorescence of 2-AP monomer probes to demonstrate the stoichiometry of gp32 binding to ssDNA lattices. It has been shown that the binding site size (*n*) for cooperatively bound clusters of gp32 is 7 nts per gp32 monomer (15), and that this site size is independent of ssDNA sequence (24). To interpret the fluorescence and CD data presented in this paper, it was necessary first to demonstrate that gp32 binds completely, cooperatively and stoichiometrically to our 2-AP-labeled ssDNA constructs under the solution conditions used in this study (see Table 1 for a listing of the nomenclature and structures of the constructs).

**Figure 1.**
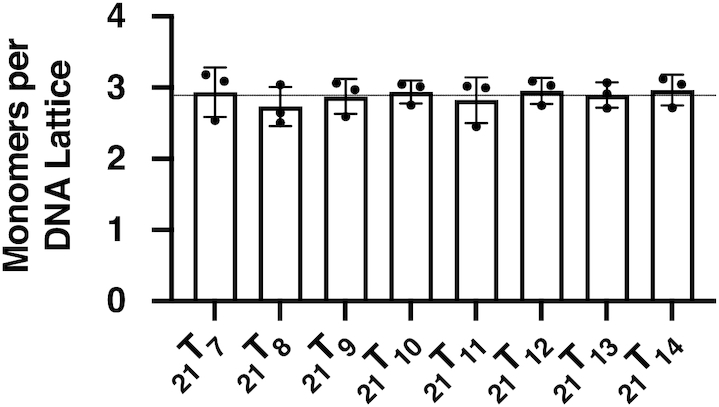
Gp32 titrations are stoichiometric and saturate at approximately 3.0 gp32 monomers per 21-mer ssDNA lattice. Summary of average gp32 saturation levels for each oligo-(dT) 21-mer ssDNA construct labeled with a 2-AP monomer probe, as monitored by fluorescence intensity change at 370 nm. Each saturation value was derived from the intersection of the slope and plateau of the relevant binding curve, as described in M&M. Saturation for each construct occurs at 2.9 ± 0.1 monomers per 21-nucleotide lattice, with no significant differences with position of the 2-AP analogue probe within the construct.

To this end, gp32 titrations were performed for all the 21-mer and the 28-mer ssDNA constructs labeled with 2-AP monomer and dimer probes that are listed in Table 1. In Supplementary Figure S1 we show, as an example, the complete set of titrations for the 21-mer (_21_T_X_) DNA constructs labeled with 2-AP in each probe position, indicating that for all these titrations, under the conditions used, binding goes to completion at stoichiometric ratios of gp32 molecules and lattice binding sites. Each panel in Supplementary Figure S1 represents the results of several (3–6) repeats of a titration with stock gp32 solution to the lattice construct at an initial concentration of 1 μM at 20°C in the ‘experimental buffer’, as described in Materials and Methods (M&M). The data were normalized as described, and dilution corrections (these corrections were relatively small, because the stock solutions of gp32 and acrylamide were quite concentrated—see M&M) were made to establish the actual ratio of gp32 monomers per 21-mer ssDNA construct at each data point. Titrations were extended well beyond the stoichiometric ‘equivalence-point’ (up to 2-fold saturation) in order to obtain accurate extrapolations, and the first and last six data points of the titration were linearly extrapolated to obtain the equivalence-point for each construct. The concentration of gp32 at which each titration reached lattice saturation averaged 2.9 gp32 monomers per ssDNA construct for all the 21-mer lattices listed in Table 1. The average of the measured intercepts for each probe position ranged from 2.8 to 3.0 bound gp32 monomers per 21-mer oligo-dT construct, as summarized in Figure 1.

We note that the use of these relatively short (finite) oligo-dT lattices as binding targets also weakens—relative to long lattices—the effective binding affinity per protein molecule for short clusters of cooperatively bound protein ligands such as gp32 (25,26). For long ssDNA lattices the effective binding constant per cooperatively bound gp32 monomer is *K*_a_ω, where *K*_a_ is the association constant per gp32 monomer and ω is the cooperativity parameter for the binding interaction (24). However, for short lattices the effective binding constant per gp32 monomer is decreased. Thus for gp32, with a binding site size (*n*) of 7 nts/gp32 monomer, the net binding (association) constant per bound gp32 molecule for fully saturated 21-mer lattices is *K*_a_ω^0.67^. The cooperativity parameter per bound gp32 molecule, ω, is raised to the 2/3rd power (i.e., the effective binding cooperativity is decreased, although the cooperativity parameter itself is unchanged) for the 21-mer lattices because at saturation each lattice binds three gp32 monomers, while the bound trimeric gp32 cluster has only 2 binding interfaces (25).

Similarly, the net association constant to the 28-mer lattices per cooperatively bound gp32 monomer is *K*_a_ω^0.75^, with the cooperativity parameter for the 28-mer lattices being raised here to the 3/4th power because the four molecule gp32 cluster bound to that lattice at saturation contains four gp32 binding sites, but only three gp32–gp32 interfaces. Thus the affinity per bound gp32 monomer at cooperative saturation would be expected to be somewhat weaker for the 21-mer lattices than for the 28-mer lattices (and of course also weaker for both constructs than the affinity per gp32 molecule bound in long clusters to long lattices). However, since the titrations for all the 21-mer constructs (see Supplementary Figure S1) show—under the solution conditions used in this study—that binding is already fully stoichiometric (i.e. manifesting a sharp break at 1:1 ratios of gp32 molecules to ssDNA binding sites) for the 21-mer (three binding site) lattices, this is certainly true for the 28-mer lattices as well. The significance of these binding free energy differences as a consequence of such ‘finite lattice’ effects have been discussed in earlier work on this system (27). Also no significant ‘end effects’ (12) should be observed at the 2-AP probe positions studied here, because at saturation the probes are all located within, or adjacent to, ‘interior’ gp32 binding sites on the ssDNA lattices.

### The fluorescence intensity for each 2-AP monomer probe position within the ‘central’ binding site of the 21-mer constructs increases 4 to 5-fold at gp32 binding saturation

We used the 21-mer oligo-(dT) ssDNA lattices listed in Table 1 to map the changes in fluorescence intensity of the 2-AP probe as a function of position within the binding cleft upon the addition of saturating concentrations of gp32. These constructs each bind (cooperatively) a cluster of three gp32 molecules at lattice saturation, and the sites labeled with 2-AP monomer probes are all within (positions 8–14), or immediately adjacent to (position 7), the ‘central’ gp32 binding site of the saturated construct. The final (plateau) fluorescence intensity at 370 nm was measured for all _21_T_X_ lattices at an initial construct concentration of 1μM in the presence of over-saturating amounts of gp32 (up to a final concentration of 5 gp32 monomers per lattice, or 4.45μM after correcting for dilution). Over-saturating amounts of gp32 were used to be sure that any errors in defining the initial concentration of each _21_T_X_ construct, as well as any inactive (for binding) gp32 protein, would not affect the outcome. We note that the monomer probes at all positions within the _21_T_X_ constructs exhibited approximately the same fluorescence intensity in the absence of gp32 (Supplementary Figure S2).

When gp32 was added to each construct an overall increase in the peak 2-AP fluorescence – to about 4 times the intensity observed with the free lattice – was observed. These major gp32-induced increases in the intensity of the probe fluorescence likely reflect primarily the fluorescence enhancement (‘unquenching’) that accompanies the unstacking of the 2-AP probes from their neighboring bases as a consequence of the extension and ‘straightening’ of the sugar-phosphate backbone of the ssDNA lattice that results from gp32 binding.

### The fluorescence intensities of 2-AP monomer probes in the 21-mer ssDNA lattice constructs at saturating gp32 concentrations differ with probe position within the binding cleft

**Figure 2.**
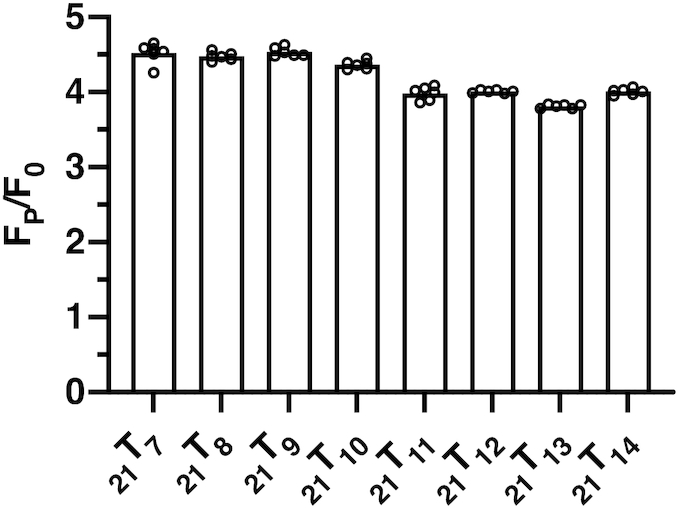
Fluorescence maxima at different 2-AP probe positions saturate at different fluorescence intensity ratios. Fluorescence intensity ratios (F_P_ is the fluorescence intensity at over-saturating gp32, F_0_ is the intensity of the construct in the absence of gp32) for 21-mer ssDNA at saturating gp32 concentrations differ with 2-AP probe position. All constructs used exhibited essentially the same degree of fluorescence in the absence of gp32, but show differences in fluorescence intensities with the addition of saturating concentrations of gp32. The points in each construct column represent the saturation fluorescence ratios of the individual titrations. Error bars for the raw data for these titrations are shown in the right-hand panel of Supplementary Figure S2.

Figure 2 shows that the final fluorescence intensity at saturating gp32 concentrations differs significantly—and beyond the limits of error—from one probe position to another, suggesting that these differences might be used to map position-specific aspects of the detailed interactions of local amino acid residues of the gp32 binding cleft with nearby elements of its ssDNA binding partner. These differences in the fluorescence intensity at the 370 nm peak are plotted as *F*_P_/*F*_0_ ratios, where *F*_0_ and *F*_P_ are the peak intensities for each probe position in the absence of gp32 protein, or in the presence of over-saturating gp32 concentrations (5 gp32 monomers per 21-mer lattice), respectively. Figure 2 shows that the observed final intensity ratios vary between 3.7 and 4.5, depending on the location of the 2-AP probe on the lattice, and thus presumably on its local environment within the binding cleft. The lattice constructs with the probe positioned near the 5′-end appear to show higher fluorescence intensity ratios than do probes located near the 3′-end of the construct, with the fluorescence intensity ratios for constructs _21_T_7_ through _21_T_10_ clustering at about 4.5, while constructs _21_T_11_ through _21_T_14_ show intensity ratios at or below 4.0, perhaps reflecting less unstacking of the adjacent nucleotide bases located within the binding cleft at these probe positions and/or smaller quenching effects due to local protein-induced polarity differences within different portions of the ssDNA binding cleft (see below and Discussion).

### Acrylamide quenching of 2-AP monomer probes differs with probe position and gp32 concentration

**Figure 3.**
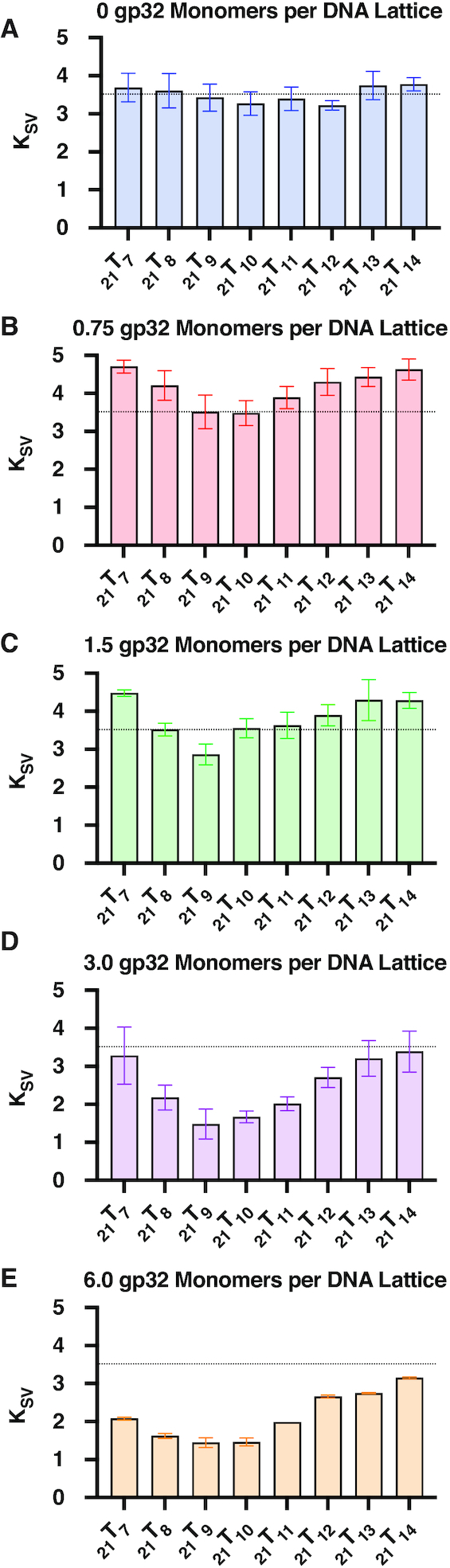
Acrylamide quenching by gp32 as a function of 2-AP monomer probe position. Titrations with monomeric acrylamide quench 2-AP fluorescence and can be used to monitor the solvent accessibility of each 2-AP probe. Calculations using the Stern–Volmer equation provide collisional quenching constants for each 2-AP monomer-probe-labeled ssDNA construct as a function of gp32 monomers added per 21-mer lattice. In each panel the horizontal dotted line marks the average *K*_sv_ value (∼3.5, see Panel A) for the probe positions of the _21_T_X_ construct in the absence of gp32 protein.

Acrylamide fluorescence quenching experiments were performed to evaluate the differences in solvent accessibility of the 2-AP base analogues in our _21_T_X_ constructs as a function of probe position and gp32 concentration. The resulting spectra were corrected as described in Materials and Methods and Stern–Volmer (S–V) quenching constants (*K*_sv_) were determined for each trace. Figure 3 summarizes the average quenching obtained from four to eight replicate experiments at each probe position and gp32 concentration, and a typical set of S–V titrations are shown in Supplementary Figure S3. The parameters obtained from S–V titrations for all of the acrylamide quenching experiments are summarized in Supplementary Table S1.

As Figure 3A shows, in the absence of gp32 the *K*_sv_ parameter obtained for each construct is essentially independent of probe position, although a slight apparent dip—which may be within the limits of error—is seen for the probes located near the middle of the central gp32 binding site. Progressive changes in *K*_SV_, and thus in 2-AP probe accessibility to the acrylamide quencher, are seen in Figure 3B–E as the ratio of gp32 monomers added per ssDNA lattice increases toward, to, and then beyond saturation.

In Figure 3B, which shows data for the sample containing 0.75 gp32 monomers per 21-mer lattice (25% saturation), we see that the probe positions located near both ends of the middle gp32 binding position show an increased quenching constant (i.e. more 2-AP probe exposure than in the control in the absence of protein), while the exposure of the probes at positions 9 and 10 (close to the 5′-end of the middle binding site) are essentially unchanged from their values in the absence of protein. In Figure 3C (half-saturation—1.5 gp32 monomers per 21-mer lattice) the end positions still show increased probe exposure relative to the controls, while position 9, in particular shows a measurable decrease in solvent accessibility. In Figure 3D (full saturation—3.0 gp32 monomers per 21-mer lattice), all positions show some degree of decreased probe accessibility, with this effect being maximal (exposure decreased to less than half of the control value) at position 9 and its immediate neighbors. In Figure 3E (twice the saturation concentration), the binding profiles are similar to those obtained at stoichiometric gp32 levels, but solvent access to all probe positions is reduced somewhat further.

A preliminary interpretation of these changes, which is consistent with previous single-molecule studies from our laboratory (28), is that at 25% saturation protein binding is quite dynamic (28), with gp32 binding as monomers to (and dissociating from) the ssDNA lattice at random. This is consistent with the notion that the ssDNA bases are being transiently unstacked and restacked as a consequence of these rapid association-dissociation processes, thus increasing the average exposure of the base analogue probes to the solvent. This tendency continues, although to a somewhat lesser extent, at 50% saturation, while at 100% saturation and above, where gp32 binds cooperatively (and as full trimer gp32 clusters) to all three lattice binding positions and exchange rates with free gp32 protein are much slower (**28**, and B. Israels et al., unpublished data), access for the acrylamide quencher to all the 2-AP probes is decreased, with the maximum decrease centered on positions 9 and 10— i.e., close to the 5′-end of the central binding site. More detailed structural interpretations of these changes are considered in the Discussion.

### CD spectral changes for ssDNA constructs site-specifically labeled with 2-AP dimer probes indicate preferential 5′-end binding in cooperative gp32 binding modes

**Figure 4.**
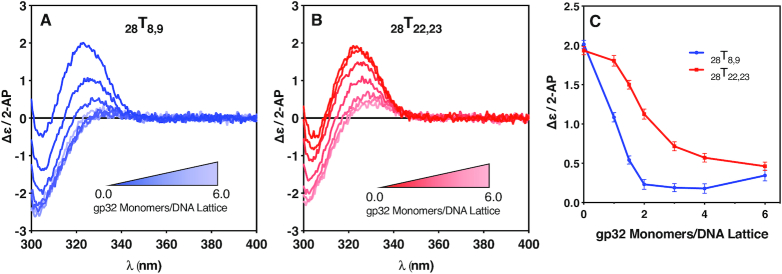
Circular dichroism changes with gp32 addition for the _28_T_x,x_ constructs. CD changes in the spectra of the 28-mer (containing four full length gp32 binding sites) 2-AP dimer-probe-labeled ssDNA constructs as a function of gp32 addition. (**A**) CD spectra of the _28_T_8,9_ construct as a function of gp32 concentration; (**B**) CD spectra of the _28_T_22,23_ construct as a function of gp32 concentration; (**C**) The Δϵ/2-AP change at 325 nm for the exciton peak of the 2-AP dimer probes for the _28_T_8,9_ construct (blue, solid circles) and _28_T_22,23_ construct (red, solid squares) over the course of the titration.

**Figure 5.**
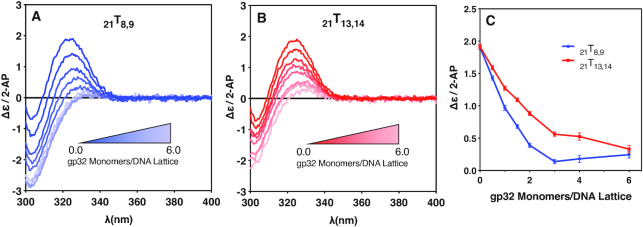
Circular dichroism changes with gp32 addition for the _21_T_x,x_ constructs. CD changes in the spectra of the 21-mer (containing three full length gp32 binding sites) 2-AP dimer-probe-labeled ssDNA constructs as a function of gp32 addition. (**A**) CD spectra of the _21_T_8,9_ construct as a function of increasing gp32 concentration; (**B**) CD spectra of the _21_T_13,14_ construct as a function of gp32 concentration; (**C**) The Δϵ/2-AP change at 325nm for the exciton peak of the 2-AP dimer probes for the _21_T_8,9_ construct (blue, solid circles) and _21_T_13,14_ construct (red, solid squares) over the course of the titration.

Circular dichroism experiments using 2-AP dimer probe pairs provide yet another approach to mapping the conformational changes that occur in the ssDNA lattice during titration with gp32, as shown in Figures 4 and 5. As the bottom four entries in Table 1 show, these constructs were 21-mer and 28-mer oligo(dT) lattices labeled with dimer probes at the 8, 9, as well as at the 13, 14 positions, and the 8, 9 as well as the 22, 23 positions (for the _21_T_XX_ and the _28_T_XX_ constructs, respectively). This places the dimer probes at the 5′ and the 3′ ends of the central (at binding saturation) gp32 binding site on the 21-mer lattices, and at the 5′ ends, respectively, of the second and fourth binding sites of the 28-mer lattices, thus reflecting conformational changes that occur at probes located at ‘internal’ positions within cooperatively bound gp32 clusters. As expected from earlier studies (12), Figures 4 and 5 confirm that—at the protein and DNA concentrations and solution conditions used in this experiment—gp32 does bind stoichiometrically to all the ssDNA lattices used, with saturation achieved at a concentration ratio of 4 gp32 molecules per 28-mer ssDNA lattice construct, and 3 gp32 molecules per 21-mer lattice.

Figure 4A shows the CD spectral changes of the 2-AP dimer probes of the _28_T_8,9_ construct that accompany the addition of increasing concentrations of gp32, up to a saturation ratio (defined as the number of gp32 monomers added per available gp32 binding site) of 1.5. In this construct the 2-AP dimer pair probe is located at the 5′-end of the second gp32 binding site on the ssDNA lattice. The intensity (at ∼325 nm) of the peak of the CD signal for the 2-AP dimer probe is largest in the complete absence of gp32. As the gp32 concentration was increased from 0 to ∼2 gp32 monomers per ssDNA lattice, exciton coupling between the 2-AP fluorophores of the dimer probe (and thus the intensities of the resulting 2-AP CD signals) were significantly reduced. As the gp32 concentration was further increased (to saturation) at 4.0, and then up to a 1.5-fold excess of gp32 (6.0 gp32 monomers per ssDNA lattice), the amplitude of the 2-AP CD peak does not decrease significantly further, with the value of Δϵ/2-AP remaining at (or slightly above) a plateau value of ∼0.2 (see Figure 4C).

Figure 4B shows the CD spectral changes of the _28_T_22,23_ construct, in which the 2-AP dimer probe is located at the 5′ end of the fourth potential gp32 binding site of the ssDNA lattice. The intensity of the CD signal contributed by the 2-AP dimer probe pair is again largest for the free ssDNA construct, as demonstrated by the darkest red spectrum (corresponding to zero gp32 monomers/DNA lattice at a 6 μM concentration of DNA construct). As the gp32 concentration increased from 0 to 4.0 gp32 monomers per DNA lattice, exciton coupling and the resulting 2-AP CD signal were again significantly reduced. As the gp32 concentration was increased to 6.0 gp32 monomers per DNA lattice (a 50% excess of gp32), the 2-AP CD signal does not change significantly further, here reaching a final value of Δϵ/2-AP ∼ 0.5.

A comparison between the titration profiles of the two lattices is shown in Figure 4C, where the amplitude of the ∼325 nm peak for each spectrum is plotted as a function of the concentration of gp32 monomers per DNA lattice, and the effects described above can be seen more clearly. As the CD-detected saturation ratio is increased from 0 to 2.0 gp32 monomers per DNA lattice, the _28_T_8,9_ plot exhibits a steep rate of decrease in the amplitude of CD spectral peak at 325 nm with increasing gp32 concentration, whereas the _28_T_22,23_ plot shows an initial ‘lag’ in the decrease of the CD peak amplitude. As the saturation ratio reaches 2.0, and then is increased further, the intensity of the CD peak for _28_T_8,9_ construct appears to plateau, while that of the _28_T_22,23_ peak continues to decrease, although more gradually.

A detailed comparison of the titration CD spectra of the two constructs is informative with respect to the local DNA conformations in the vicinity of the spectral probes. The decrease in the height of the spectral peak at about 325 nm for both constructs presumably reflects the unstacking and separation of the bases of the dimer pair probe with increasing gp32 concentration, as also suggested by the fluorescence amplitude increases of the monomer 2-AP probes monitored in Figure 2. We can estimate from the lengthening of the ssDNA lattice with added gp32 (to ∼4.6 Å/nt for ssDNA lattices fully saturated with gp32 [see (16)]) that at saturation the centers of the two 2-AP bases of the dimer probe are separated by 1 to 2 Å relative to their separation in the no-protein ssDNA control, where the bases of the ssDNA construct are largely stacked in an average conformation close to that of the B-form Watson-Crick conformation (i.e., with an average rise per residue of ∼3.4 Å per base) in the absence of gp32.

This is consistent with the interpretation that the 2-AP bases are progressively unstacked and separated as gp32 binds, but also – since the height of the CD spectral peak at 325 nm (especially for the _28_T_8,9_ construct) actually approaches zero – suggests that the chirality of the ssDNA helix may also be approaching zero, meaning that the ssDNA backbone of the constructs is likely close-to-fully linearly extended in this region of the titration curve for the _28_T_8,9_ construct. Comparison with the titration curves for the _28_T_22,23_ construct (Figure 4B) shows that for the latter probe-pair this backbone stretching and straightening (and therefore the loss of exciton coupling) does not go as far, suggesting that some chirality of base stacking (and exciton coupling between the 2-AP bases of the dimer probe) may be retained in this portion of the binding cleft of a gp32 molecule located at the 3′-end of the lattice, even though at this point both constructs are saturated with gp32 (see Figure 1). This difference between the titration profiles of the two 28-mer constructs is clearly seen at the higher CD-detected saturation ratios in Figure 4C as well. This suggests, at minimum, that gp32 binding results in somewhat different conformational changes in ssDNA lattice positions at comparable positions in the binding cleft of gp32 molecules located, respectively, near the 5′-end and near the 3′-end of the lattice.

Related CD results were obtained for gp32 titrations of the 21-mer oligo(dT) constructs with site-specifically placed 2-AP dimer probes at the two ends of the ‘middle’ gp32 binding site, as shown in Figure 5. Here (see Table 1) we utilized two 21-mer lattices containing a 2-AP dimer probe pair located either at the ‘beginning’ of the second binding site (_21_T_8,9_), or at the ‘end’ of the second binding site (_21_T_13,14_). The results also demonstrate major changes in the CD peak intensity at ∼325 nm with increasing gp32 concentrations. Figure 5A shows the CD spectral changes for the _21_T_8,9_ construct, with the 2-AP dimer probe pair located at the 5′-end of the central gp32 binding site. Again, the amplitude of the CD peak contributed by the 2-AP dimer probe pair is greatest for the construct in the absence of added gp32. As the gp32 concentration is increased from 0 to 2.0 gp32 monomers per DNA lattice, the amplitude of the CD peak due to the 2-AP dimer probe is significantly reduced, although not as steeply as the decrease observed with the _28_T_8,9_ construct. Starting at a gp32 concentration of 3.0 gp32 monomers per lattice, and up to a two-fold over-saturation concentration of 6.0 gp32 monomers per lattice, the amplitude of the 2-AP CD peak does not appear to change significantly further, and again appears to plateau at a CD intensity of ∼0.2 Δϵ/2-AP.

Figure 5B shows the CD spectral changes for the _21_T_13,14_ construct, which has its 2-AP dimer probe pair located near the 3′-end of the middle binding site, and also closer to the 3′-end of the 21-mer ssDNA lattice. As in Figure 5A, the amplitude of the CD peak is largest in the absence of gp32 (the ‘construct alone’ signal) at a DNA concentration of 6 μM. As the protein concentration is increased from 0 to 2.0 gp32 monomers per protein binding site, the amplitude of the CD peak of the 2-AP dimer probe is significantly reduced, although with a rate of intensity decrease with increasing gp32 less than that seen with the _21_T_8,9_ construct. As the gp32 concentration approaches binding saturation (at 3.0 gp32 monomers per DNA lattice), and then reaches twice saturation (at 6.0 gp32 monomers per DNA lattice), the intensity of the dimer-probe CD signal changes only minimally. Comparison of the results with the two lattices are shown directly in Figure 5C, where the ∼325 nm peak of each spectrum for each lattice is plotted as a function of gp32 monomers per DNA lattice. As the gp32 concentration is increased from 0 to 2.0 gp32 monomers per DNA lattice, the _21_T_8,9_ plot shows a faster rate of amplitude decrease than does the peak obtained with the _21_T_13,14_ construct. As the gp32 concentration ratio reaches 3.0, and then is increased to 6.0 gp32 monomers per DNA lattice, with gp32 binding again reaching and then surpassing the saturation ratio of 3 gp32 monomers per DNA lattice, both plots appear to ‘bottom out’ at values of Δϵ/2-AP less than 0.5.

We note that, for both the 28-mer and the 21-mer ssDNA lattices, the conformational changes monitored by the intensity of the CD peak for the dimer 2-AP probes go to completion at lower gp32 concentrations for the dimer probes located closer to the 5′-ends of the constructs than for the dimer probes located closer to the 3′-ends of the constructs. This effect is more obvious for the 28-mer than the 21-mer, as expected because binding is more cooperative for the longer lattice. Because gp32 binding to both of these constructs is significantly cooperative, at partial (i.e., half) saturation levels of gp32 about half of the ssDNA lattices will be close to fully saturated, while the other half will have bound little or no gp32. Since the effective binding cooperativity per bound gp32 monomer for the 28-mer lattices will be greater than for the 21-mer lattices (see above), this would suggest that the rate of change of Δϵ/2-AP in Figure 4C should be somewhat greater than in Figure 5C, and this is consistent with what we see in comparing these two data sets.

## DISCUSSION

### Overview

An aim of this work was to extend our earlier studies (6,12) of the interactions of a minimally perturbing base analogue probe with gp32 to all positions within the ssDNA binding cleft of a T4 ssb protein bound cooperatively to the ssDNA lattice. Such ‘saturation mapping’ allows us to characterize the conformational changes and interactions of our base analogue probes at every lattice position with the locally apposing (and interacting) amino acid residues that make up the walls of the binding cleft. We have used three complementary spectroscopic approaches that take advantage of different spectral properties of site-specifically placed monomer or dimer 2-AP base analogue probes at defined positions within a ssDNA lattice otherwise consisting entirely of dT residues. We note that these approaches can, of course, be used in various combinations for solution studies of a wide variety of DNA-protein (and RNA-protein) interactions.

- *2-AP fluorescence enhancement induced by gp32 binding*. Our first approach measured the enhancement of 2-AP fluorescence induced by local interactions at each monomer probe position within the ssDNA footprint of cooperatively bound gp32. Such fluorescence enhancement (without significant peak shift) as a consequence of protein binding is often seen and has been widely used to monitor local changes in protein-DNA interactions (6,12,29–31). We show in this study that the fluorescence amplitude of the probe at each position within a ssDNA lattice otherwise consisting solely of dT residues (and also within lattices of mixed base composition in earlier studies (12)) is essentially the same, indicating that by this criterion the unperturbed ssDNA lattice has, on average, a fairly uniform conformation. Under our experimental conditions, the ssDNA bases likely fluctuate non-cooperatively around a largely stacked Watson-Crick B-form conformation with an average right-handed helical pitch of ∼3.4 Å per nucleotide residue (32, also E. Beyerle et al, unpublished results).
  The very large (4- to 5-fold) increase in fluorescence observed at all ssDNA lattice positions as a consequence of gp32 binding (Figure 2) is consistent with the fact that this binding significantly unstacks the bases at all positions within the gp32 footprint and that such unstacking significantly reduces the quenching effect of nearest neighbor bases on the fluorescence of the 2-AP monomer probe, even for relatively weakly stacked neighboring dT residues. This major fluorescence increase is likely a consequence of the lattice-extending activity of the gp32 protein, which involves some straightening of the sugar-phosphate backbone of the ssDNA, presumably with a concomitant partial untwisting of the ssDNA helix and the separation of adjacent (and initially stacked) DNA bases. The differences in the magnitude of this effect as a function of probe position may reflect minor differences in the extent of this unstacking and untwisting at different lattice positions within the gp32 binding site, but may also involve differences in local polarity or dielectric constant that are induced by the proximity of the different amino acid residues that line the cleft.
- *Acrylamide quenching studies*. A second, and complementary, spectroscopic approach that we have used in this study and previously (6,12,20) involves dynamic (Stern-Volmer) quenching of the fluorescence of the position-specific 2-AP monomer probes by solvent additives. Here we use acrylamide monomers as uncharged quenchers (rather than the usual iodide or cesium ionic quenching agents that are often used in protein studies) to avoid coulombic interactions with the ssDNA backbone phosphate groups. Acrylamide appears to act as a standard collisional (diffusion-limited) quencher with our 2-AP base analogue probes, yielding linear Stern-Volmer plots for our acrylamide titrations (see Supplementary Figure S3 and Results). The Stern–Volmer parameters (K_SV)_ obtained from the slopes of these plots provide information about differences in solvent access to the 2-AP monomer probes as a function of lattice position, both in the absence and the presence of cooperatively bound gp32 trimeric clusters on our probe-labeled 21-mer oligo(dT) lattice constructs.
  We find that, in the absence of protein, acrylamide access to all lattice probe positions is – like 2-AP fluorescence enhancement – essentially probe-position independent. However, as shown in Figure 3 and described in Results, access of the acrylamide quencher to 2-AP base probes near the middle of the central gp32 binding site is significantly reduced relative to access to the probes at the ends of the central gp32 footprint, especially in the presence of saturating concentrations of cooperatively bound gp32.
- *Circular dichroism changes with increasing gp32 concentrations*. Finally, following up on earlier studies with ssDNAs of mixed base composition (6,12), we have monitored changes in the CD spectra of 2-AP dimer probes positioned in otherwise purely oligo(dT) lattices at or near the two ends of ‘internal’ gp32 binding sites as a function of increasing gp32 concentrations, and have observed significant differences in how the gp32 binding cleft interacts with these dimeric (and internally exciton-coupled) probes, both initially in the titrations where gp32 binds largely at random as rapidly-exchanging monomers, and then at saturating protein levels where gp32 binds cooperatively as stoichiometrically bound protein ‘clusters’. The resulting CD spectra monitor differences in the exciton coupling of the 2-AP dimer probes at defined positions within the binding cleft at various stages of gp32 binding saturation, and show (see Figures 4 and 5) that—even at saturation—the detailed conformations of the ssDNA nts bound within the binding cleft differ from one position to another.

### Comparison with structural implications from X-ray crystallography

An early X-ray diffraction study of the DNA binding domain of gp32 co-crystallized with a short (6-mer) ssDNA lattice provides some hints toward possible structural interpretations of some of the spectroscopic observations in this paper and its predecessors (6,12). However, these interpretations must be viewed with caution because (perhaps due to relatively weak and somewhat labile binding of this short lattice within the crystal) the structural details of the bound ssDNA chain could not be resolved in the X-ray study (5), although the presence the ssDNA oligonucleotide in the gp32 binding cleft (and its general path through the cleft) could be surmised from the extra electron density due to the physical presence of the ssDNA in the binding cleft.

**Figure 6.**
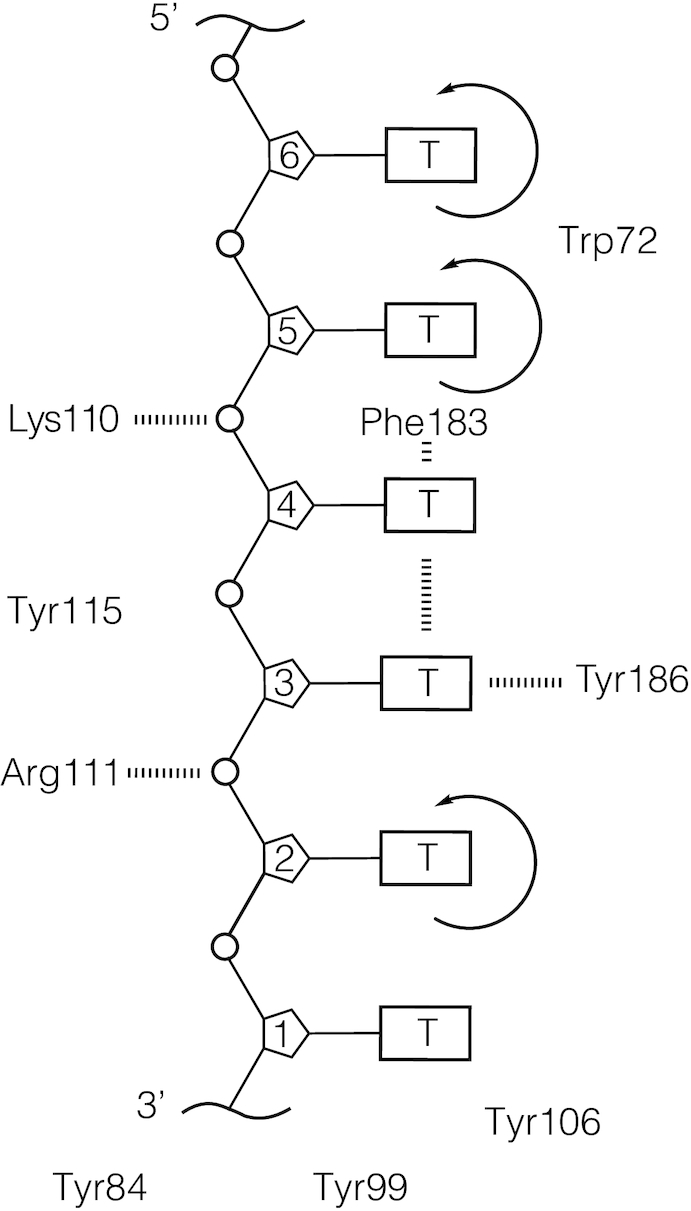
Schematic of possible local amino acid and nucleotide residue interactions within the binding cleft of the gp32 ‘core’ domain, based on the Shamoo et al. crystal structure (5). Electron density maps of core gp32 bound to a dT_6_ ligand were obtained at 2.2 Å resolution (Shamoo *et al.* 1995). While the overall electron density of the ligand was rather weak, some suggestions were made from the partially resolved structure. These are summarized in this schematic. The authors suggested that electrostatic interactions occur between the phosphate oxygens of nucleotide residues T2 and T4 and amino acids Arg111 and Lys110, respectively. Furthermore, residues T3 and T4 appear to form a stacked conformation, which may also include Phe183. Nucleotide residue T3 also appears to be aligned in an end-on-end interaction with Tyr183. The bases of residues T2, T5 and T6 are described as being translationally and rotationally unconstrained within the model, with residues T5 and T6 appearing to lie in the vicinity of Trp72.

In Figure 6, we provide a speculative schematic diagram showing possible interactions with the DNA chain of some of the residues that line the binding cleft, as positioned in the Shamoo et al. study (5) and based on suggestions by those authors as to how these residues might interact with the bound ssDNA lattice. We then attempt to consider whether—and to what extent—these suggestions are consistent with our spectroscopic results. The limited resolution of the ssDNA lattice in the crystal structure may be due in part to the fact that the interactions of the protein with the ssDNA lattice are largely electrostatic, and involve direct binding of charged amino acid residue side-chains to the sugar-phosphate backbone of the ssDNA lattice, while the DNA bases protrude from the groove and are largely available to interact with other replication proteins. This may well have made it possible for the bound dT_6_ chain to ‘slide’ somewhat in the gp32 binding cleft, even within the crystal, and thus may have ‘blurred’ the electron density of the dT_6_ ligand itself, although crystal packing forces could limit such motions. Nevertheless, this blurring likely also made it impossible for Shamoo et al. to determine the binding polarity of the oligonucleotide ligand relative to the structure of the gp32 DNA binding domain.

However, as described in our earlier spectroscopic studies of this system (6,12), we have been able to infer that the ssDNA lattice binds in the cleft with its 5′-end close to the position at which the N-terminal domain emerges from the DNA binding domain, and its 3′-end near where the C-terminal domain emerges from the DNA binding domain. Thus we orient the 6-mer section of the dT_6_ lattice shown in Figure 6 with that polarity, and describe (below and in the figure legend) some of the possible interactions of the amino acid residues of the cleft with specific nucleotide residue positions along the chain.

Shamoo et al. suggested from their study that tetranucleotide 1–4 of the dT_6_ lattice (see Figure 6), and in particular residues 2 and 3, could be fairly well localized within the gp32 core domain and thus might be more tightly bound than residues 5 and 6, which could not be localized. This is consistent with earlier results (9) and with our previous 2-AP studies (6,12), which suggested that gp32 monomers bind most tightly to the sugar-phosphate backbone of a short ssDNA lattice at positions close to the 3′-end of the chain (i.e. at positions 2–3 in the schematic in Figure 6). The crystallography suggested that the positions of the dT bases attached to sugars 2, 5 and 6 might be relatively unconstrained, while the phosphates between sugar 5 and 4 and between sugar 3 and 2 might interact electrostatically with positively-charged Lys 110 and Arg 111, respectively.

Shamoo et al. also suggested that the aromatic residues Trp 72, Phe 183 and Tyr 186 might also interact (perhaps via some form of ‘incomplete stacking’) with the nucleotide positions indicated in the schematic in Figure 6, and that the dT bases in positions 3 and 4 appeared to be rather tightly stacked on one another, while Phe 183 might be somewhat stacked with the dT residue located in position 4. They also suggested that Trp 72 might be rather close to the dT bases at positions 5 and 6. We note that there is some ambiguity in the numbering system used in the schematic of Figure 6, due to the fact that the site-size of the actual binding footprint of a gp32 monomer is **7** nts. Thus, we do not know whether the 6 dT residues shown in Figure 6 correspond to residues 1–6 or to residues 2–7 of the ‘real’ gp32 monomer footprint.

### Structural implications from our spectroscopy studies

In our earlier study of the binding of gp32 monomers to a 2-AP-labeled dT_8_ ligand (12), we concluded that these monomers bind initially, and most tightly, to two nucleic acid backbone positions near the 3′-end of the ssDNA lattice (perhaps equivalent to positions 2 and 3 in Figure 6), with other positions binding somewhat more weakly – perhaps due in part to the partial ‘blocking’ of those regions of the lattice by one of the possible binding conformations of the C-terminal domain. (This domain, of course was not present in the ‘core’ DNA binding domain studied by Shamoo et al.). These (and earlier) results also suggested that the direct binding interactions of monomer-sized lattices with the gp32 binding cleft are essentially the same in the DNA binding domain and in the full gp32 monomer (with the C-terminus ‘swung out of the way’—see Figure 6 of Jose *et al.* (12)). We note that the direct binding affinity (*K*_a_) for a cooperatively bound gp32 monomer (as studied here and in (12)) is approximately the same for the free monomer and the cooperatively-bound monomer, although of course the total binding affinity (K_a_ω) for a cooperatively bound gp32 monomers is increased by the contribution of ω, the cooperativity parameter. These findings are also consistent with the larger fluorescence enhancement (less stacking, presumably accompanied by more solvent exposure) seen in Figure 2 with 2-AP monomer probes near the 5′-end of the cooperatively bound lattice, and with the decreased access to the acrylamide quenching agent (presumably due to decreased solvent exposure) of 2-AP probes located near the 5′-end of the lattice, as seen in Figure 3.

### Applicability of these complementary spectroscopic approaches to other systems

In conclusion, and as suggested above, these approaches can clearly all be generalized to map significant aspects of DNA–protein interactions in a variety of biologically interesting processes involved in genome expression. Using such different spectroscopic methods in combination increases the power of this approach since—as pointed out above—the three methods used here are significantly differentially sensitive to local variations in ssDNA conformation within a protein–DNA binding complex. Thus a comparative study of this sort can provide detailed and unique information about lattice structure and dynamics at different positions within the binding clefts of DNA-binding proteins that is not available from the averaged binding parameters (ligand binding site size, binding affinity and binding cooperativity) that are obtained by straight-forward thermodynamic measurements (24,25).

## Supplementary Material

gkaa1230_Supplemental_File

## Notes

Present address: Katarina Meze, W.M. Keck Structural Biology Laboratory, Cold Spring Harbor Laboratory, Cold Spring Harbor, NY 11724, USA.

Present address: Anson Dang, Johns Hopkins University, Thomas C. Jenkins Department of Biophysics, 110 Jenkins Hall, 3400 N. Charles Street, Baltimore, MD 21218, USA.
