# Supplementary material for: Mapping DNA conformations and interactions within the binding cleft of bacteriophage T4 single-stranded DNA binding protein (gp32) at single nucleotide resolution
Source: Nucleic Acids Res. 2020 Dec 24;49(2):916–27. doi: 10.1093/nar/gkaa1230 (PMC7826291; doi:10.1093/nar/gkaa1230)
Supplement: gkaa1230_Supplemental_File [file gkaa1230_supplemental_file.pdf]

Supplementary Information for NAR paper by Camel et al.

Mapping DNA conformations and interactions within the binding cleft of bacteriophage T4 single-stranded DNA binding protein (gp32) at single nucleotide resolution.

Benjamin R. Camel[1], Davis Jose[1,2]*, Katarina Meze[1,3], Anson Dang[1,4] and Peter H. von Hippel[1]*

[1] Institute of Molecular Biology and Department of Chemistry and Biochemistry, University of Oregon, Eugene, OR 97403, USA.

[2] Department of Chemistry and Physics, Monmouth University, West Long Branch, NJ 07764, USA.

[3] Present address: W.M. Keck Structural Biology Laboratory, Cold Spring Harbor Laboratory, Cold Spring Harbor, NY 11724, USA.

[4] Present address: Johns Hopkins University, Thomas C. Jenkins Department of Biophysics, 110 Jenkins Hall, 3400 N. Charles Street, Baltimore, MD 21218, USA.

*Co-corresponding authors. e-mail addresses: D.J., djose@monmouth.edu; P.H.v.H., petevh@uoregon.edu.

Figure S1. Binding stoichiometry gp32 titrations for the 21-mer ($_{21}T_x$) DNA constructs for each probe position. Binding stoichiometry titrations for each oligo-dT 21-mer ssDNA construct labeled with a 2-AP monomer probe, as monitored by fluorescence intensity change at 370 nm. Each data set was individually fit using a linear extrapolation for both the slope and plateau of the binding curve, as described in the **Materials and Methods**.

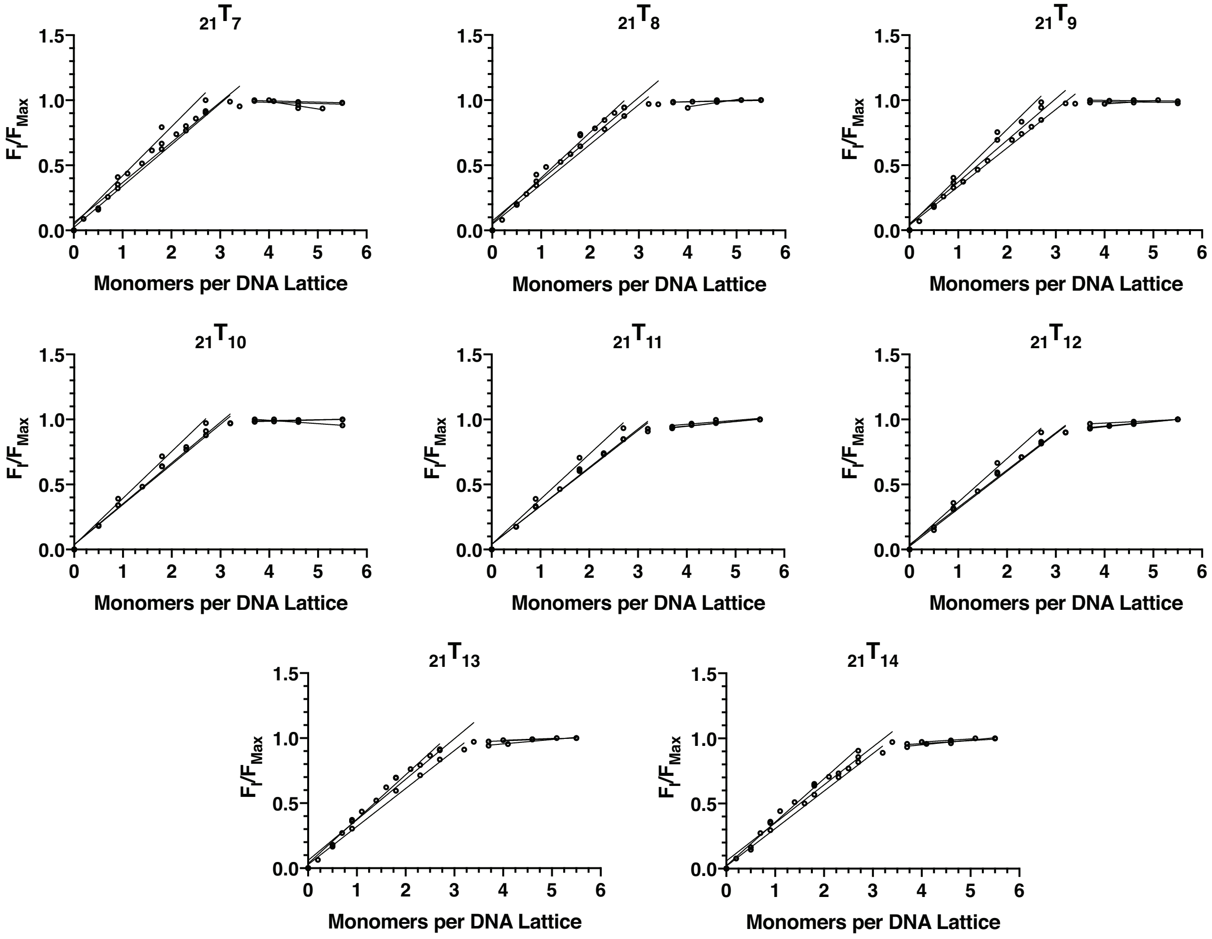

Figure S2. Fluorescence intensities of the 2-AP monomer probes at the 370 nm emission peak for each $_{21}T_x$ construct in the absence and presence of saturating amounts of gp32. Fluorescence intensities for each oligo-(dT) 21-mer ssDNA construct labeled with a 2-AP monomer probe, in the absence (white bars) or the presence (grey bars) of saturating concentrations of gp32. Constructs in the absence of gp32 show closely similar fluorescence intensities, regardless of probe position. With the addition of saturating concentrations of gp32, fluorescence intensities increase overall and show significant differences as a function of probe position, as shown more clearly in **Figure 2** of the main text.

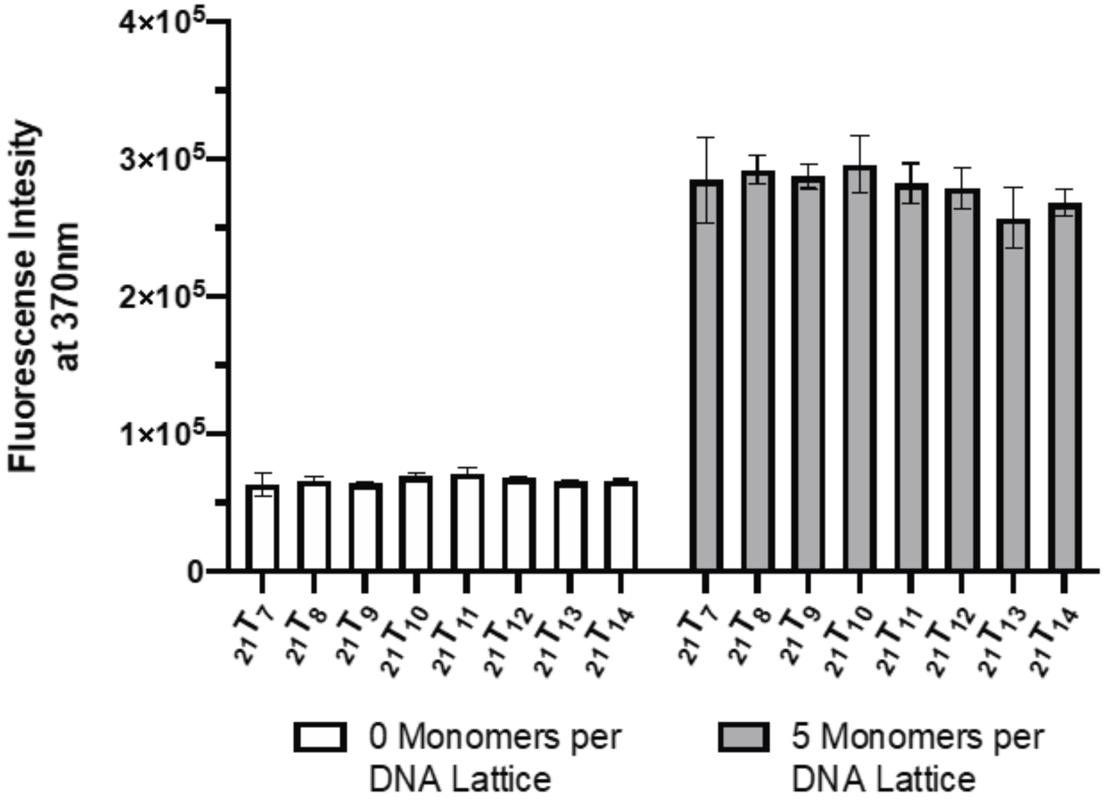

Figure S3. Stern-Volmer plots for each 2-AP monomer probe-labeled 21-mer ($_{21}T_x$) ssDNA construct equilibrated with varying gp32 concentrations and titrated with monomeric acrylamide as a collisional quenching agent. Titrations were performed in the absence of gp32, and with 0.75, 1.5, 3.0, and 6.0 gp32 monomers per ssDNA construct. These results are for the titrations made for gp32 concentrations of 1.5 gp32 monomers per lattice. Linear regressions are shown for each replication titration.

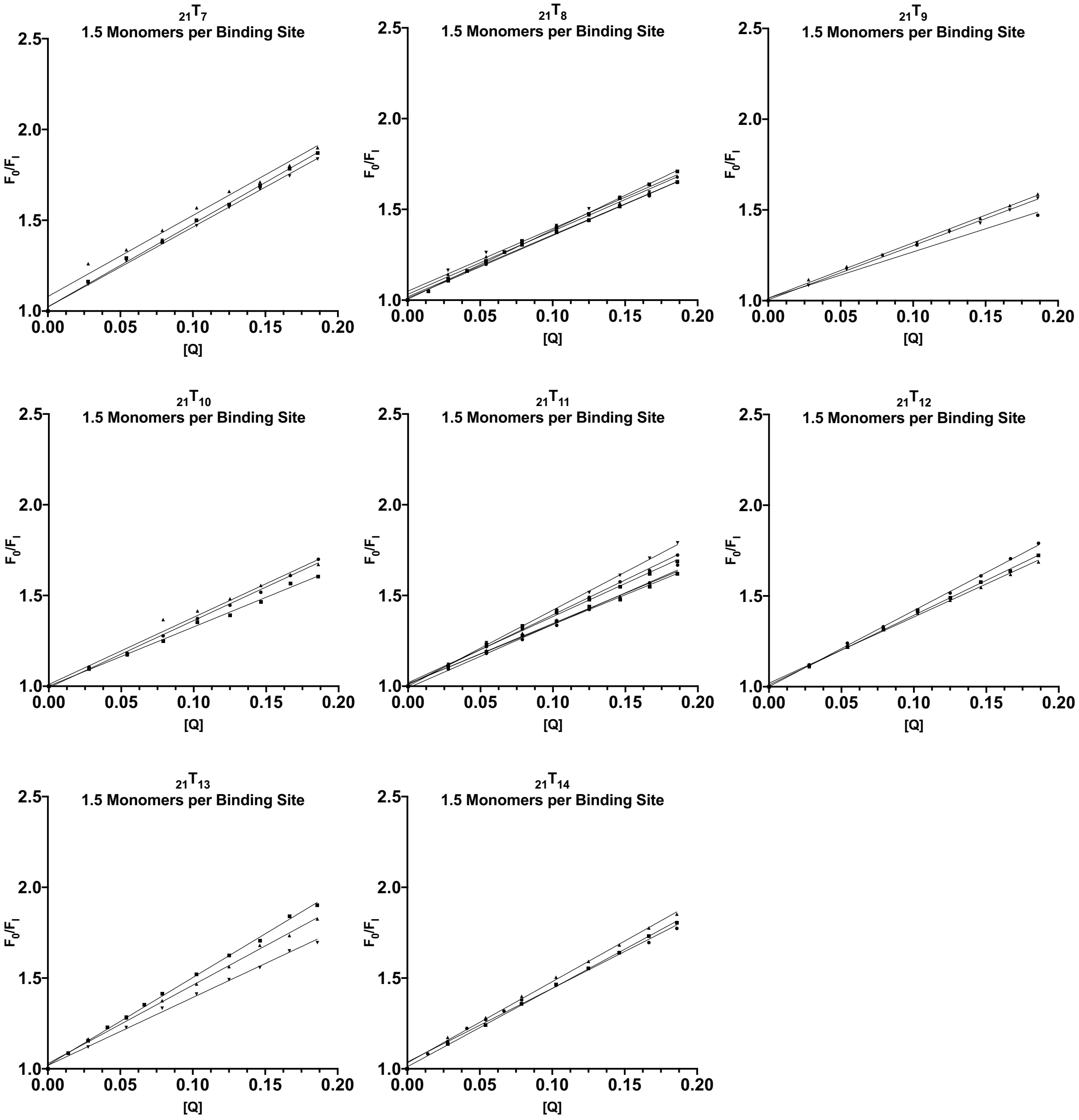

Table S1. Summary of Stern-Volmer titration fit parameters. Summary table showing the K_{SV} values and their respective error (standard deviation) obtained for each $_{21}T_x$ construct at various concentrations of gp32, as described in **Materials and Methods** and illustrated in **Figure S3**.

Table S1: Average Stern-Volmer constant and error for each 2-AP monomer probe-labeled 21-mer ssDNA constructs with varying gp32 concentration

	DNA Alone			0.75 Monomers per DNA Lattice			1.5 Monomers per DNA Lattice			3.0 Monomers per DNA Lattice			6.0 Monomers per DNA Lattice		
**$_{21}T_7$**	3.69	±	*0.37*	4.71	±	*0.17*	4.48	±	*0.08*	3.28	±	*0.75*	2.08	±	*0.04*
**$_{21}T_8$**	3.61	±	*0.45*	4.21	±	*0.39*	3.52	±	*0.17*	2.18	±	*0.33*	1.62	±	*0.07*
**$_{21}T_9$**	3.42	±	*0.36*	3.51	±	*0.44*	2.86	±	*0.27*	1.48	±	*0.40*	1.44	±	*0.13*
**$_{21}T_{10}$**	3.27	±	*0.31*	3.48	±	*0.33*	3.55	±	*0.25*	1.67	±	*0.15*	1.46	±	*0.10*
**$_{21}T_{11}$**	3.39	±	*0.31*	3.89	±	*0.29*	3.63	±	*0.35*	2.01	±	*0.18*	1.98	±	*0.00*
**$_{21}T_{12}$**	3.22	±	*0.12*	4.30	±	*0.35*	3.90	±	*0.28*	2.71	±	*0.26*	2.66	±	*0.04*
**$_{21}T_{13}$**	3.74	±	*0.37*	4.44	±	*0.25*	4.29	±	*0.54*	3.21	±	*0.47*	2.75	±	*0.02*
**$_{21}T_{14}$**	3.78	±	*0.18*	4.63	±	*0.28*	4.28	±	*0.21*	3.39	±	*0.54*	3.15	±	*0.03*
